# The Effect of Carbohydrate Ingestion on Performance during a Simulated Soccer Match

**DOI:** 10.3390/nu5125193

**Published:** 2013-12-16

**Authors:** Julia H. Goedecke, Nicholas J. White, Waheed Chicktay, Hafsa Mahomed, Justin Durandt, Michael I. Lambert

**Affiliations:** 1 UCT/MRC Research Unit for Exercise Science and Sports Medicine, University of Cape Town, P.O. Box 115, Newlands, 7725, Cape Town 7700, South Africa; E-Mails: nickwhitebio@gmail.com (N.J.W.); wchicktay@gmail.com (W.C.); Mike.lambert@uct.ac.za (M.I.L.); 2 South African Medical Research Council, Parow, Cape Town 7505, South Africa; 3 UCT/MRC Research Unit for Nutrition and Dietetics, Department of Human Biology, University of Cape Town, Cape Town 7700, South Africa; E-Mail: hafsamahomed@gmail.com; 4 Discovery Health High Performance Centre, Sports Science Institute of South Africa, Newlands, Cape Town 7700, South Africa; E-Mail: JDurandt@ssisa.com

**Keywords:** intermittent exercise, supplementation, sports drinks, football

## Abstract

Aim: This study investigated how performance was affected after soccer players, in a postprandial state, ingested a 7% carbohydrate (CHO) solution compared to a placebo (0% CHO) during a simulated soccer match. Methods: Using a double-blind placebo-controlled design, 22 trained male league soccer players (age: 24 ± 7 years, wt: 73.4 ± 12.0 kg, VO_2_max: 51.8 ± 4.3 mL O_2_/kg/min) completed two trials, separated by 7 days, during which they ingested, in random order, 700 mL of either a 7% CHO or placebo drink during a simulated soccer match. Ratings of perceived exertion (RPE), agility, timed and run to fatigue were measured during the trials. Results: Change in agility times was not altered by CHO *vs*. placebo ingestion (0.57 ± 1.48 *vs*. 0.66 ± 1.00, *p* = 0.81). Timed runs to fatigue were 381 ± 267 s *vs*. 294 ± 159 s for the CHO and placebo drinks, respectively (*p* = 0.11). Body mass modified the relationship between time to fatigue and drink ingestion (*p* = 0.02 for drink × body mass), such that lower body mass was associated with increased time to fatigue when the players ingested CHO, but not placebo. RPE values for the final stage of the simulated soccer match were 8.5 ± 1.7 and 8.6 ± 1.5 for the CHO and placebo drinks respectively (*p* = 0.87). Conclusions: The group data showed that the 7% CHO solution (49 g CHO) did not significantly improve performance during a simulated soccer match in league soccer players who had normal pre-match nutrition. However, when adjusting for body mass, increasing CHO intake was associated with improved time to fatigue during the simulated soccer match.

## 1. Introduction

Soccer is defined as an intermittent sport characterised by periods of high-intensity play in addition to periods of sub-maximal effort over ±90 min. Players cover approximately 9–12 km per game, depending on their position and level of play [1,2]. The average intensity during a match is about 70%–75% of the VO_2_max, with much of the energy demand coming from sprinting and other motions such as dribbling, heading, tackling and running backwards and sidewards [1,3]. The causes of fatigue during soccer are complex, and may involve various mechanisms acting centrally and peripherally [4]. However, one of the main mechanisms of fatigue during a soccer match is the depletion of liver and muscle glycogen [5,6].

Many studies, which have examined the effect of carbohydrate (CHO) ingestion during simulated/real life soccer matches, have been comprehensively reviewed by Phillips *et al*. [7]. These studies have mostly used the Loughborough Intermittent Shuttle test (LIST), which elicits similar demands to a soccer match [8,9]. The players in most of these studies ingested 5 mL/kg of a 6%–7% CHO solution prior to exercise, and 2 mL/kg every 15 min during exercise [7]. In general, these studies have found that CHO ingestion before and during exercise reduces muscle glycogen utilization [10,11], maintains plasma glucose levels [12], improves running time to fatigue [13,14,15] and coordination [12,14] in the latter stages of the exercise trial. The effects on sprinting times were less consistent, with only a few studies showing improvements in sprinting performance [12,14]. More recently, the effects of CHO ingestion on simulated soccer performance have been undertaken in adolescents, the results being similar to those in adults, showing an overall improvement in time to fatigue [16,17].

In their review, Phillips *et al*. [7] concluded “*early research was almost unanimous in supporting the consumption of carbohydrate-electrolyte solutions during prolonged intermittent exercise for maintaining and/or improving exercise performance and capacity*”. However, the authors also concluded that many of the studies had methodological concerns that limited their applicability to actual team games. Notably, several studies have tested participants in a fasted state [12,13,14], or have not reported on the nutritional status of the participants [16,17]. This detracts from the interpretability of the data because in a real-life situation, players are not usually fasted before a match. Further, in a match situation, it is difficult to regulate the exact volume of fluid required by each player. Therefore our goal was to use a similar research design to what has been used previously [8], but to focus on the fact that the players started the trial in the same fed state as they would be before a match and each received the same volume of fluid throughout the simulated soccer match.

Therefore, the aim of the study was to determine whether ingestion of 700 mL of a 7% CHO solution, ingested immediately before and during the Loughborough intermittent shuttle test, would improve agility and running time to fatigue in league soccer players who had normal pre-match nutrition.

## 2. Methods

### 2.1. Participants and Study Design

Twenty-two male soccer players, currently involved in competitive local league soccer were recruited for the trial. After being informed of the nature of the study, participants gave their written consent to participate. The Human Research Ethics Committee of the Faculty of Health Sciences at the University of Cape Town approved the study.

In this randomized, double-blind, placebo-controlled trial, participants completed two trials, separated by 7 days. During the trials, participants ingested in random order either a commercially available 7% CHO sports drink or a placebo beverage (0% CHO) of similar taste and electrolyte concentration. All trials were held at approximately the same time of day, to negate any diurnal effect on results. The participants were instructed to prepare for the trial, from a nutrition and exercise perspective, as they would normally prepare for a soccer match, in an attempt to simulate pre-match conditions. However, they were asked to refrain from eating 2 h before the testing session to avoid any gastro-intestinal side effects.

**Figure 1:**
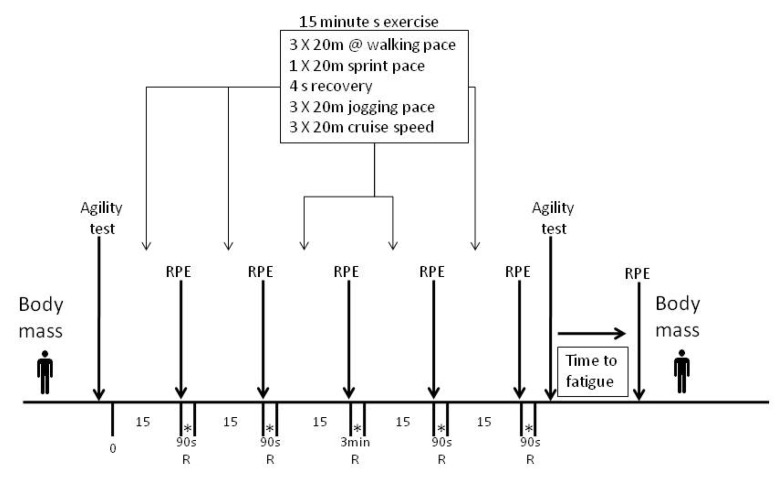
Schematic representation of the simulated soccer match adapted from the modified Illinois agility test [18] and the Loughborough Intermittent Shuttle Test (LIST) [8]. RPE, rating of perceived exertion; R, rest; * ingested 50 mL.

During each trial, participants completed a previously validated simulated soccer match, followed by a run to fatigue (LIST) [8] and a modified Illinois agility test [18]. Body mass was measured pre- and post-exercise to estimate sweat losses. Participants recorded their ratings of perceived exertion [19] after every exercise bout, as well as following the run to fatigue. The design is summarised in Figure 1.

### 2.2. Familiarisation

Before the study, participants completed a familiarisation session during which body mass, height and skinfold thickness (bicep, triceps, sub scapular, suprailiac) were measured. Body fat percentage was estimated using the equations of Durnin and Womersley [20]. Participants then completed a multi-stage shuttle run to estimate their maximal oxygen uptake capacity (VO_2_max) [21]. The average VO_2_max of the team was then used to calculate the speeds at which participants ran during the subsequent testing sessions in the LIST. To familiarise themselves with the testing protocol, each subject completed at least two 15-min bouts of the intermittent shuttle test.

### 2.3. Dietary Intake

Participants were instructed to eat as they would normally do in preparation for a soccer match and to replicate the same dietary strategy before each trial. Participants recorded their intake on the day leading up to each trial. The dietary records were analysed for total energy and macronutrient content using Foodfinder^®^ Version 3 (National Nutritional Intervention Unit, MRC, Parow, Cape Town, South Africa, 2002).

### 2.4. Exercise Trials

On arrival at the laboratory, the pre-test body mass of the participants was measured. They then completed a 15 min standardised warm-up of stretching and jogging. Participants performed the pre-trial modified Illinois Agility test [18] to assess soccer-specific agility. During this test each participant dribbled a soccer ball through the cones set up according to a standard Illinois agility run. The participants were electronically timed during this test (Powertimer, Powertimer technology, Newtest Oy, Tyrnävä, Finland) to enhance accuracy. Each player did the test twice within a 15 min period, and the best score was recorded. The test was repeated if the participant lost control of the ball during the test.

Participants then completed the LIST [8], designed specifically to simulate soccer match conditions. The LIST was modified slightly to enable a team of players to be tested at the same time, and to allow time to complete the soccer-modified Illinois agility test prior to and following the LIST. The total time of the revised protocol, including rest breaks, was 90 min. The test is divided into 2 parts. The first part consisted of 5 periods of 15 min of exercise, with each period separated by a 90 s break and a 3 min half-time break after the 3rd period. Each 15 min period consisted of the following pattern of repeated exercise, with the running speeds of 55% and 95% being calculated from the group predicted VO_2_max using tables for predicted VO_2_max values [21]:

3 × 20 m at a walking pace (1.54 m/s/12.98 s for 20 m)
1 × 20 m sprint pace (6.2 m/s/3.22 s for 20 m)
4 s recovery
3 × 20 m at a jogging pace at 55% of the group-predicted VO_2_max (3.00 m/s)
3 × 20 m at cruise speed, at 95% of the group-predicted VO_2_max (3.83 m/s)

This pattern was repeated approximately 11 times in the 15-min period. The running and walking speeds for each 20 m were dictated by an audio CD designed for this purpose. Participants then completed the soccer-modified Illinois agility test again. The change in performance in the agility test over the trial was thus determined.

Following the agility test, participants completed a timed run to fatigue, which consisted of 20 m runs at progressively increasing speeds. Fatigue was described as the point where the participant could not complete two consecutive shuttles.

The body mass of each participant was measured again, and the change in body mass over the trial was used as an indicator of sweat loss. The calculation considered the fluid ingested during the trial. Heart rate was recorded continuously throughout the trial using heart rate monitors (Suunto T6, Suunto, Amer Sports Corporation, Helsinki, Finland). The players’ level of fatigue was measured using a visual analogue scale according to the Borg [19] following every 15-min exercise session, following the agility tests and following the completion of the run to fatigue.

### 2.5. Drink Administration

During each trial, participants ingested 700 mL of either a commercially available sucrose-based 7% CHO sports drink (Energade, Tiger Consumer Brands Ltd., Bryanston, Johannesburg, South Africa) or an artificially flavoured placebo beverage (0% CHO) of identical electrolyte concentration (sodium 41 mg/100 mL, potassium 5 mg/100 mL) and similar colour and taste.

Each participant ingested 250 mL of the randomly assigned drink, before the warm up and following the third 15-min exercise bout. In addition, 50 mL of the drink was ingested during the 90 s break separating each 15-min exercise bout. Consequently, participants ingested 49 g CHO during the CHO trial and no CHO during the placebo trial.

Participants completed a post-test drink survey to assess their perceptions of the drink (overall taste, after-taste, sweetness and level of stomach fullness or discomfort) on a visual analogue scale. The participants were also asked questions regarding the perceived performance benefits of the drinks, as well as possible side effects, and if they would use the drink during a soccer match in the future. 

### 2.6. Statistical Analyses

Sample size was determined based on the findings of Welsh *et al*. [14] and Nicholas *et al*. [13]. Using a power of 80% and an α of 0.05, with effects sizes for the differences between CHO and placebo trials of 0.69 and 0.59, respectively, one would require a sample of 17–23 participants.

Data are presented as mean ± standard deviation. Dependent t-tests were used to compare dietary intake before the two simulated soccer trials. Two-way ANOVA with repeated measures were used to measure differences over time between drinks with respect to heart rate, agility and rating of perceived exertion, with a Tukey HSD *post-hoc* analysis. Friedman ANOVA was used to analyse non-parametric data (subjective perception of drinks). ANOVA and ANCOVA, adjusting for body mass or body fat %, were used to compare the change in heart rate, RPE, agility, and time to fatigue of the players when ingesting CHO or placebo. To determine whether differences in body mass (or body fat %) of the players influenced the main outcome measures (time to fatigue and change in agility), multiple regression was used including drinks, body weight (or body fat %) and their interaction (drink × body weight (or body fat %)) as independent variables in the model. The Cohen’s effect size was calculated to quantify the magnitude of difference between trials for the outcome measures of agility and time to fatigue. The following nomenclature of categorization was used: trivial (ES ≤ 0.2), small (ES = 0.2 to 0.5), medium (ES > 0.5 to 0.8) [22]. Statistical significance was accepted as *p* < 0.05. Data were analysed using STATISTICA version 10 (Statsoft Inc., Tulsa, OK, USA) and STATA SE version 12.1 (StataCorp, College Station, TX, USA).

## 3. Results

The average age, body mass, height and body fat of the 22 soccer players was 24 ± 7 years, 73.4 ± 12.0 kg, 171.4 ± 4.7 cm and 16% ± 5%, respectively. Their mean VO_2_max, estimated in a progressive shuttle run test, was 51.8 ± 4.3 mL O_2_/kg/min.

### 3.1. Dietary Intake

There was no significant difference in dietary intake in the day preceding the CHO and placebo trials, with the majority of energy intake being derived from CHO (Table 1).

**Table 1:** Average energy and macronutrient intake 1 day prior to the carbohydrate (CHO) and placebo trials.

	Placebo trial	CHO trial	*p* Value
Energy (kJ)	6834 ± 3723	6335 ± 3043	0.621
CHO (g)	209 ± 130	190 ± 104	0.533
CHO (%)	51.4 ± 14.7	51.3 ± 14.0	0.994
Protein (g)	60 ± 36	53 ± 20	0.441
Protein (%)	17.6 ± 14.9	16.5 ± 10.5	0.770
Fat (g)	53 ± 27	55 ± 34	0.856
Fat (%)	29.3 ± 7.5	31.0 ± 10.3	0.561

Values are mean ± SD.

### 3.2. Heart Rate and Rating of Perceived Exertion (RPE)

**Figure 2:**
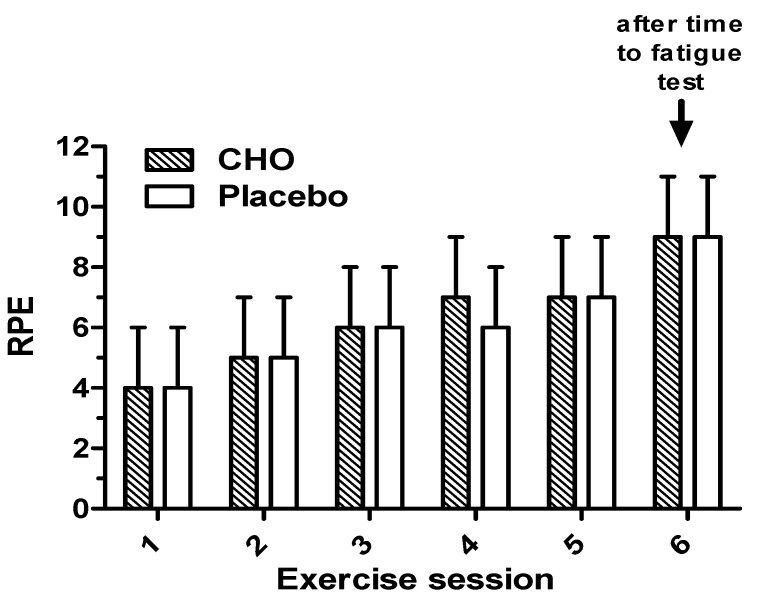
Changes in RPE during the simulated soccer match while ingesting the placebo (0% CHO) and 7% CHO solutions.

Average and maximal heart rates recorded during the simulated soccer trial were not different between trials (Ave: 151 ± 13 *vs*. 150 ± 15 bpm, *p* = 0.813; Max: 192 ± 16 *vs*. 188 ± 13 bpm, *p* = 0.268, for placebo and CHO trials, respectively). RPE increased significantly following each 15 min stage of the simulated soccer match (*p* < 0.05), with a further increase following the run to fatigue (*p* < 0.05) (Figure 2), but there were no differences between CHO and placebo trials (*p* = 0.97). Adjusting for body mass or body fat % did not alter the findings.

### 3.3. Performance

**Figure 3:**
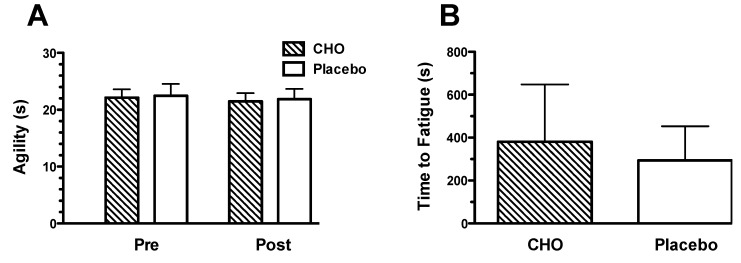
Changes in agility (**A**) and the time to fatigue (**B**) during the simulated soccer match while drinking the placebo (0% CHO) and 7% CHO solutions.

**Figure 4:**
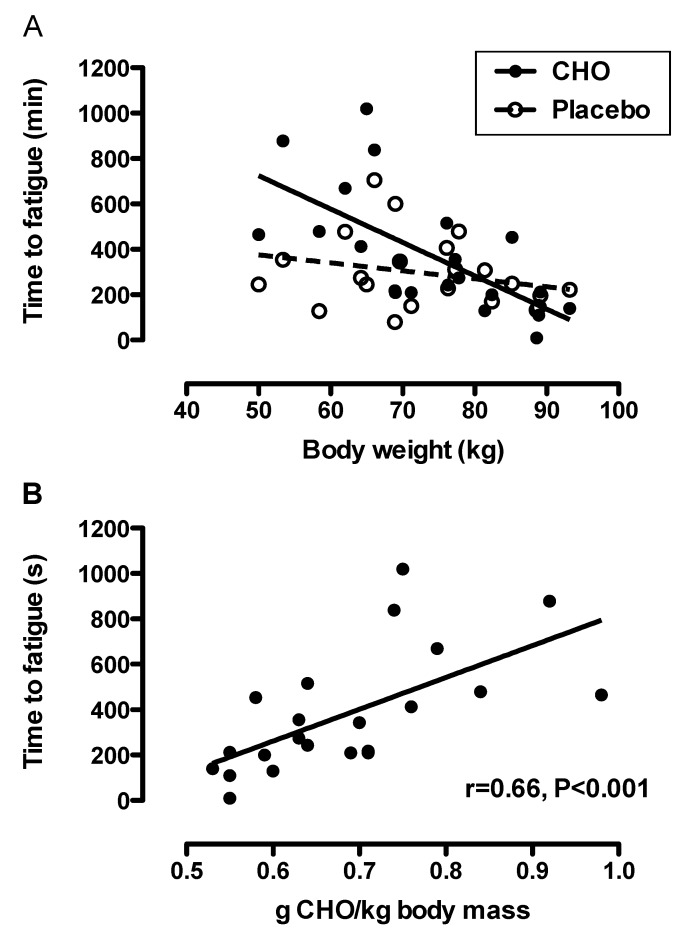
(**A**) The influence of body mass on time to fatigue during the simulated soccer match while ingesting the placebo (0% CHO) or the 7% CHO solutions (*p* = 0.023 for drinks × body mass interaction). (**B**) The relationship between CHO ingestion (g/kg body mass) and time to fatigue during the simulated soccer match.

Agility and time to fatigue were not different between the CHO and placebo trials (Figure 3), even after adjusting for body mass and body fat % (change in agility: *p* = 0.807 and *p* = 0.776; time to fatigue: *p* = 0.141 and *p* = 0.182, respectively). The effect size of the difference between the pre/post agility results (CHO *vs*. placebo) was 0.01 (*i.e.*, trivial), and the effect size of the difference between the time to fatigue (CHO *vs*. placebo) was 0.39 (*i.e.*, small). When exploring whether differences in body mass (or body fat %) of the players influenced the performance outcomes, we found that body mass (but not body fat %, *p* = 0.368) modified the relationship between time to fatigue and drink ingestion (*p* = 0.023 for drink × body weight interaction), such that lower body weight was associated with increased time to fatigue when the players ingested CHO (*r* = −0.66, 95% CI: −0.85 to 0.33, *p* < 0.001), but not placebo (*r* = −0.26 95% CI: −0.62 to 0.18, *p* = 0.235) (Figure 4). No interaction effects between body mass or body fat % and drink ingestion was found for change in agility (*p* = 0.118 and *p* = 0.462, respectively).

### 3.4. Palatability of Drinks and Sweat Loss

The participants’ perception of taste, after-taste, sweetness and fullness between the CHO and placebo drinks were not different (Table 2). There was no difference in sweat loss between the two trials, with the average change in body mass over the trial being 0.90 ± 0.43 *vs*. 0.88 ± 0.37 kg (*p* = 0.875) for the placebo and CHO trials, respectively.

**Table 2:** Palatability of CHO and placebo drinks.

	Placebo trial	CHO trial	*p* Value
Taste (mm)	6.8 ± 2.4	7.7 ± 1.8	0.11
After-taste (mm)	2.8 ± 2.4	3.6 ± 2.6	0.26
Sweetness (mm)	5.1 ± 2.1	5.6 ± 1.9	0.38
Fullness (mm)	2.8 ± 2.1	3.5 ± 2.6	0.20

Values are mean ± SD.

## 4. Discussion

Although many studies have examined the effects of CHO ingestion on simulated soccer performance (reviewed by [7]), most of these studies have tested participants who have started the trial in a fasted state. This research design has a low ecological validity, reducing the applicability of the results to real-life situations. Accordingly, we designed our study to mimic a real match situation as much as possible. The participants were encouraged to prepare as they would before a typical league match, including normal pre-match nutrition strategies. With this research design we showed that the ingestion of a 7% CHO drink prior to, and during exercise, did not alter the group ratings of perceived exertion, heart rate response, agility, or running time to fatigue during a simulated soccer match. However, when examining the effects of body mass on performance, we found that the lighter players benefited more from CHO ingestion than the heavier players (Figure 4A). Increasing CHO intake (per kg body mass) was associated with increased time to fatigue during the simulated soccer match. 

The improvement in time to fatigue in our study and others [13,14] could be attributed to the effect of CHO ingestion on fuel metabolism during the 90 min simulated soccer match [4]. Indeed, studies have found that CHO ingestion before and during exercise reduces muscle glycogen utilization [10,11] and maintains plasma glucose levels [12]. The results of our study are somewhat surprising, as it has been shown previously that CHO ingestion improves 1 h exercise performance when CHO stores are sub-optimal, but not when the athletes start the trial in a CHO replete state [23]. In contrast, Krustrup *et al*. [6] showed that despite ingesting breakfast, as well as a meal 2 h prior to a friendly soccer match, muscle glycogen decreased significantly, with 47% of muscle fibres being completely or almost depleted of muscle glycogen after the game, Further, they showed that reduced muscle glycogen stores were associated with reduced performance in the latter stages of the soccer match, even though these friendly matches were performed at a lower intensity than those reported for elite competitive games [6]. More recently, Souglis *et al*. [24] showed that ingestion of a high CHO diet (8 g/kg body mass) for 3 days prior to a soccer match increased the distance covered (from easy jogging to sprinting) compared to a low CHO diet (3 g/kg body mass). The improvement in performance with CHO ingestion *vs*. placebo (when controlling for bodyweight) in our study could not be attributed to differences in CHO intake on the day leading up to the simulated soccer match, as their CHO intake was remarkably similar (209 *vs*. 190 g for the placebo and CHO trials respectively). However, the effects of CHO ingestion prior to and during the simulated soccer match may have been accentuated due to the players’ relatively low CHO intake on the day prior to the trial (<3 g/kg body weight).

During the trial, the participants drank a constant volume of CHO. However, this resulted in varying intake of CHO/kg body mass, ranging from 0.53 to 0.98 g CHO/kg body mass. This affected the time to fatigue of the players, such that those who received more CHO/kg had a greater improvement in performance than those who received less CHO/kg. The participants in our trial ingested 49 g CHO during the 90 min simulated soccer trial (28 g/h, including the 15 min warm-up period), which just falls outside the recommendation of 30–60 g CHO/h for activities of this nature [25]. However, based on our results, this is not sufficient to enhance performance, particularly in those participants with a larger body mass. Accordingly, when the mean performance results were compared between the CHO and placebo trials, there was no significant difference, masking the ergogenic potential of CHO ingestion (Figure 3). Irrespective of body mass, we observed large variability in response to CHO ingestion, which may reflect variability in phenotype for substrate oxidation [26].

Other studies that have previously shown an improvement in running time to fatigue [10,13,14] and coordination [12,14] in the latter stages of a simulated soccer match have administered 5 mL/kg of a 6%–7% CHO solution prior to exercise, and 2 mL/kg every 15 min during exercise, providing approximately 70–80 g CHO during the 90 min trials, equivalent to approximately 0.9 g CHO/kg body mass [13,14,15]. Although the total fluid ingestion was also greater in this study than to our own (±1200 mL *vs*. 700 mL, respectively), differences in performance could not be attributed to dehydration, as changes in body mass was similar between trials (0.90 *vs*. 0.88 kg for placebo and CHO trials, respectively).

CHO ingestion did not improve agility in our study, even after adjusting for differences in body size (Figure 3). Results relating to the effects of CHO ingestion on sprint and skill performance are less consistent than those for time to fatigue, with only a few studies showing improvements in sprint performance [12,14] and soccer skills [12,14,27]. However, this may be due to methodological issues rather than the influence of CHO ingestion [7].

This study is not without limitations. In trying to make the study conditions as representative of match conditions as possible we had to compromise on certain aspects in the research design. In particular the participants ingested a constant volume of CHO during the trial. However, we believe that this design may also be viewed as a strength, as we were able to demonstrate the body mass related relationship between CHO ingestion and performance during a simulated soccer match. Although we did not individually quantify the pre-match meal, we did quantify dietary intake during the day leading up to the research trials, which was not different between trials. However, it should be acknowledged that differences in the pre-exercise meal might have influenced performance. Other strengths of the study included the randomised, double blind, placebo controlled design. We used the LIST [8], which has been shown to elicit similar physiological and metabolic response compared to a match [8,28], and also included a soccer-specific agility test [18]. The palatability of the CHO and placebo drinks and other aspects of taste were not different, therefore it is unlikely that participants could distinguish between the two drinks. The 7% CHO solution was a commercially available product, typically used by players in matches. The study had good statistical power and all participants were league soccer players and the design included all the players of two teams. However, these were league players of varying fitness (VO_2_max range from 42.4 to 58.2 mL/kg body mass), and also body composition (body fat range from 10% to 21% body fat), which increased the variability of the results. Indeed, there was large variation in time to fatigue (Figure 4), which was influenced by body mass, but may also reflect the participants’ individual responsiveness to CHO. This is a plausible explanation because of the well-known phenotypic variation that exists, which manifests as predominantly CHO burners or fat burners [26].

## 5. Conclusions

In conclusion, the group data showed that ingestion of 700 mL of a 7% CHO solution (49 g) did not significantly improve performance during a simulated soccer match in league soccer players who had normal pre-match nutrition. However, when adjusting for body mass, increasing CHO intake was associated with improved time to fatigue during the simulated soccer match. Based on these findings, the ingestion of a pre-match meal, as well as the provision of sufficient CHO, based on body size, should be emphasised during a soccer match to ensure improved performance during the latter stages of a match.
